# *In vivo *study of endothelial barrier-related GTPase expression in the kidney and liver during the acute phase of nonlethal sepsis

**DOI:** 10.1186/cc14068

**Published:** 2014-12-03

**Authors:** BC Zychar, MA Cenezede, AMA Liberatore, NOS Câmara, IH Koh

**Affiliations:** 1 Department of Surgery, UNIFESP/EPM, São Paulo, Brazil; 2 Laboratory of Cellular Immunology Experimental, UNIFESP/EPM, São Paulo, Brazil

## Introduction

Maintenance of the integrity of the endothelial barrier is crucial in pathological inflammatory/infectious conditions [1]. The endothelial barrier dysfunction leading to increased vascular permeability and leukocyte transmigration in the systemic inflammatory state has been intrinsically related to the multiple organ dysfunction syndrome. The Rho GTPases family regulates the organization of the actin cytoskeleton, and plays a fundamental role in maintaining homeostasis and function of the endothelial barrier [2]. The study of the mechanisms of intracellular signaling related to the integrity of the endothelial barrier may help in understanding the hemodynamic changes during systemic infection. Thus, this study aimed to correlate the pattern of genic expression of GTPases during the initial periods of sepsis in order to understand the kinetic of these molecular mechanisms in organs often affected in sepsis.

## Methods

Wistar rats weighting 200 to 250 g were submitted to: nonlethal sepsis (2 ml *Escherichia coli *10^7 ^UFC/ml i.v. inoculation [3,4], *n *= 5, S7 group); minor trauma (cervical incision with catheter implantation in jugular, injection of 2 ml saline, *n *= 5, SHAM group); without any procedure (*n *= 5, NAIVE group). After 2 and 6 hours, the liver and kidney were collected to determine RhoA, Rac1 and Cdc42 gene expression by quantitative real-time PCR.

## Results

**Figure 1:**
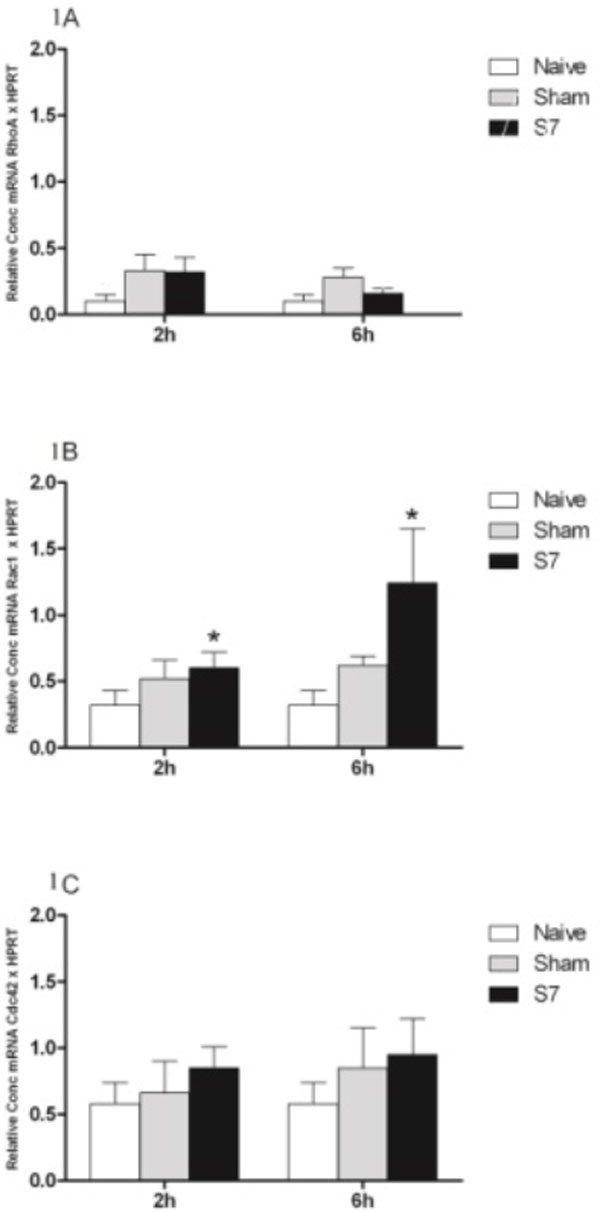
**Gene expression of GTPases RhoA **(A)**, Rac1 **(B) **and Cdc42 **(C) **in the liver of rats**. Animals were subjected to nonlethal sepsis 2 ml *Escherichia coli *10^7 ^UFC/ml i.v. inoculation (S7 group), minor trauma 2 ml saline i.v. inoculation (SHAM group), or animals without inoculation were used as control group (NAÏVE group). After 2 or 6 hours the kidney was isolated. Total RNA was extracted and transcribed to cDNA. Gene expression was quantified by real-time PCR. Graph bars show the relative concentration of each cDNA compared with the NAIVE group and the housekeeping gene HPRT. Results are mean ± SEM (*n *= 5 animals/group) and analyzed in triplicate. *P < 0.05 compared to NAÏVE group.

**Figure 2:**
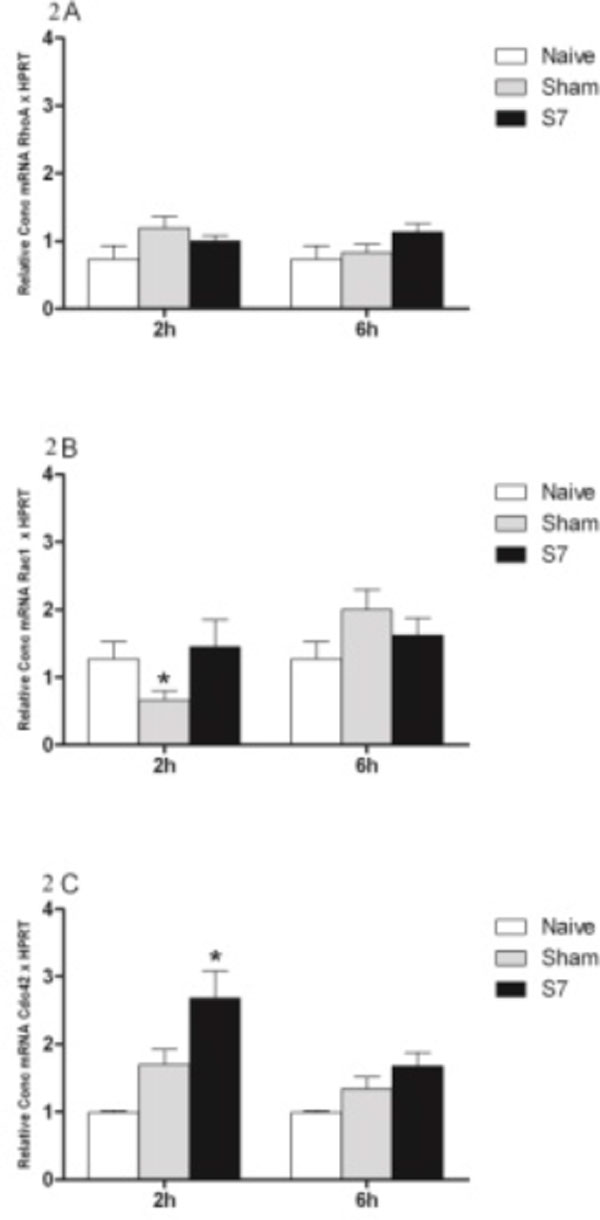
**Gene expression of GTPases RhoA **(A)**, Rac1 **(B) **and Cdc42 **(C) **in the kidney of rats**. Animals were subjected to nonlethal sepsis 2 ml *Escherichia coli *10^7 ^UFC/ml i.v. inoculation (S7 group), minor trauma 2 ml saline i.v. inoculation (SHAM group), or animals without inoculation were used as control group (NAÏVE group). After 2 or 6 hours the kidney was isolated. Total RNA was extracted and transcribed to cDNA. Gene expression was quantified by real-time PCR. Graph bars show the relative concentration of each cDNA compared with the NAIVE group and the housekeeping gene HPRT. Results are mean ± SEM (*n *= 5 animals/group) and analyzed in triplicate. *P < 0.05 compared to NAÏVE group.

Low expression of RhoA was observed in both organs (Figures 1A and Figure 2A). Rac1 and/or Cdc42 showed higher expression in both organs in animals submitted to systemic infection (S7 group) compared to the SHAM and NAÏVE groups in both periods (Figures 1B, C and Figure 2B, C). Although both organs showed a similar pattern, the kidney showed a statistically significant increase of Cdc42 and Rac1 as compared to the liver, showing that GTPase expression might differ, possibly due to endothelial heterogeneity of each organ to perform its specific function. These results suggest that the infectious process leads to a higher gene expression of Rac1 and/or Cdc42 in relation to a minor surgical trauma inflammation. The RhoA and Rac1 gene expression were inversely correlated, as reported in the literature [5].

## Conclusion

Rac1 and Cdc42 possibly act in favor of preserving the integrity of the endothelial barrier by their attributed stabilization and protection of microvessel endothelial cells, by gathering the cell junctions during the infectious state. Studies are being conducted to better understand the mechanisms of intracellular signaling of the endothelial barrier in different organs of animals submitted to varying degrees of systemic infections.
